# Functionalization of Multi-Walled Carbon Nanotubes with Thermo-Responsive Azide-Terminated Poly(*N*-isopropylacrylamide) via Click Reactions

**DOI:** 10.3390/molecules18044599

**Published:** 2013-04-18

**Authors:** Xin Su, Ya Shuai, Zanru Guo, Yujun Feng

**Affiliations:** 1 State Key Laboratory of Polymer Materials Engineering, Polymer Research Institute, Sichuan University, Chengdu 610065, China; 2 Center for Macromolecular Sciences, Chengdu Institute of Organic Chemistry, Chinese Academy of Sciences, Chengdu 610041, China; 3 University of the Chinese Academy of Sciences, Beijing 100049, China

**Keywords:** multi-walled carbon nanotubes, RAFT, NIPAM, temperature-responsive, click chemistry

## Abstract

Covalently functionalized multi-walled carbon nanotubes (MWNTs) were prepared by grafting well-defined thermo-responsive poly(*N*-isopropylacrylamide) (PNIPAM) via click reactions. First, azide-terminated poly(*N*-isopropylacrylamide) (N_3_-PNIPAM) was synthesized by reversible addition fragmentation chain-transfer (RAFT) polymerization, and then the N_3_-PNIPAM moiety was connected onto MWNTs by click chemistry. The products were characterized by means of FT-IR, TGA and TEM. The results show that the modification of MWNTs is very successful and MWNTs functionalized by N_3_-PNIPAM (MWNTs-PNIPAM) have good solubility and stability in water. TEM images show the functionalized MWNTs are dispersed individually, indicating that the bundles of original MWNTs are separated into individual tubes by surface modification with polymer chains. These MWNTs modified with PNIPAM represent a potential nano-material for preparation of hydrophilic composite materials.

## 1. Introduction

Carbon nanotubes (CNTs) [1] have attracted extensive industrial and academic attention thanks to their exciting potential applications in sensors [2], nanocomposites [3], molecular devices [4] or advanced materials with electronic properties [5]. However, their inherent insolubility in most organic and aqueous solvents, together with the poor chemical and biological compatibility of CNTs, are the major limitations to the solution-phase manipulation and processability of these structures, greatly hindering the wide application of CNTs in practical use [6].

In this respect, much effort, including noncovalent and covalent modification [7,8,9], has been made in the surface modification of CNTs, mainly to enhance their solubility and processability. The advantage of noncovalent modification is that the structure and original properties of CNTs are not altered after modification, but this method is limited to several hydrophobic polymers, such as nylon and polyacrylonitrile dissolved in toxic organic solvents, and furthermore, high concentrations of polymers are usually necessary to obtain dispersions of CNTs, and most importantly, it is difficult to further modify CNTs with different functionalities [8,9]. On the other hand, covalent sidewall modification with polymeric structures has shown promise in improving the solubility of nanotube-polymer conjugates, even with a relatively low degree of functionalization [10,11]. Furthermore, the versatility of polymer chemistry allows for control over the final properties of the nanotube-polymer hybrids, which are dictated by the chemical and physical characteristics of the grafted polymer. For example, many polymers such as polystyrene (PS) [12,13], poly(methyl methacrylate) [14,15], or polyacrylamide [16], have been used for surface modification of CNTs by covalent functionalization. Although a number of research groups have focused on functionalizing CNTs with various polymers, the ability to solubilize separate individual CNTs into water remains a great challenge.

For the applications of CNTs-based sensors and probes in a biological environment and medical chemistry, some aspects have not been explored very thoroughly, such as how to functionalize a carbon nanotube to make it water-soluble and responsive to environmental stimuli such as pH, temperature, or ionic strength [17]. These studies require CNTs that are not only water-soluble, but also well-controlled with regard to modification of the surface to obtain a shell that is sensitive to the environmental conditions [18]. Poly(*N*-isopropylacrylamide) (PNIPAM), a well-known thermo-sensitive polymer, has a low critical solution temperature (LCST) in water (around 32 °C) and represents probably the most often-used thermoresponsive polymer in biotechnology and medicine [19,20,21,22,23]. Therefore, it can be expected that CNTs will find use in new potential applications if temperature-sensitive PNIPAM chains were attached onto their sidewalls.

The covalent method for connecting PNIPAM onto CNTs must also be considered and it is another key to determine and influence the properties of CNTs. So far, covalent CNTs-polymer conjugates could be synthesized by either the “grafting from” or “grafting to” techniques [9]. The “grafting from” mechanism promises high graft densities. For instance, this technique was used to graft PNIPAM from multi-walled carbon nanotubes (MWNTs) by reversible addition−fragmentation chain transfer (RAFT) polymerization [17]. However, initiator groups are hard to attach to CNTs and the desired polymer molecular weight and architecture are difficult to achieve and control [9]. On the contrary, the “grafting to” procedure allows full control over polymer molecular weight and structure, but it suffers from low theoretical polymer loadings due to steric repulsion between grafted polymer chains. Pan *et al.* [24] used this method for grafting PNIPAM to MWNTs by thiol-coupling reactions, but the modification reaction takes more than 36 h. A more efficient coupling protocol is demanded to generate relatively high nanotube graft densities.

The copper-catalyzed azide-alkyne cycloaddition (click chemistry) discovered by Sharpless has been widely employed and confirmed to be highly efficient [25,26,27,28]. It would be very convenient and effective to graft polymer chains to CNTs by using such a coupling reaction. Adronov and co-workers pioneered the research on the linkage of polystyrene with single-walled carbon nanotubes (SWNTs) via click chemistry [29]; later, the same group of authors modified MWNTs using poly(*N*,*N*-dimethylacrylamide)-poly(*N*-isopropylacrylamide) (PDMA-PNIPAM) [30]. Cho *et al.* [31] also successfully attached PS to MWNTs by click reaction. Although fabrication of innovative materials based on MWNTs is still challenging [32], these results confirmed click chemistry is a useful tool for modifying the surface properties of CNTs with various functionalities to satisfy special end use [33].

In this work, MWNTs were modified and functionalized with the thermo-responsive homopolymer PNIPAM by click chemistry. The azide-terminated PNIPAM was prepared by RAFT polymerization with an azide-capped chain transfer agent (N_3_-CTA), and the alkyne groups were installed at the sidewalls of MWNTs. Then, the azide-terminated polymer was coupled and MWNTs were functionalized via Cu(I)-catalyzed 1,3-dipolar cycloaddition.

## 2. Results and Discussion

**Scheme 1:**
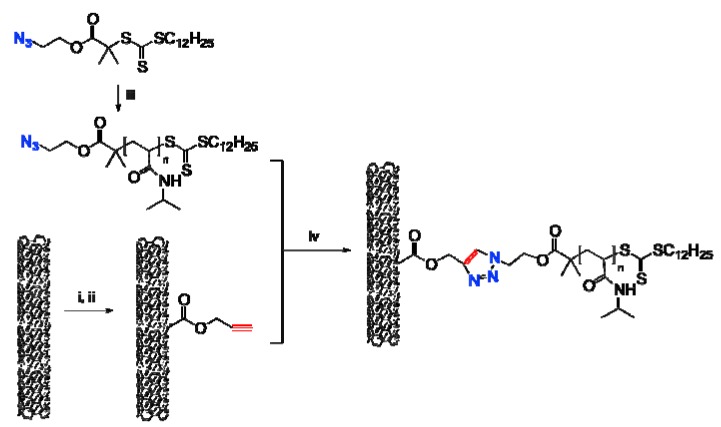
The protocol to functionalize MWNTs with PNIPAM.

Herein, we reported a two-step approach to functionalize MWNTs with a temperature-responsive polymer. As illustrated in Scheme 1, azide-decorated PNIPAM, namely N_3_-PNIPAM, was prepared through RAFT polymerization, and MWNTs were covered with alkyne groups; then the MWNTs modified with PNIPAM were obtained through coupling reaction via click chemistry.

### 2.1. Preparation of MWNTs with Alkyne Groups (MWNTs-alk)

**Figure 1:**
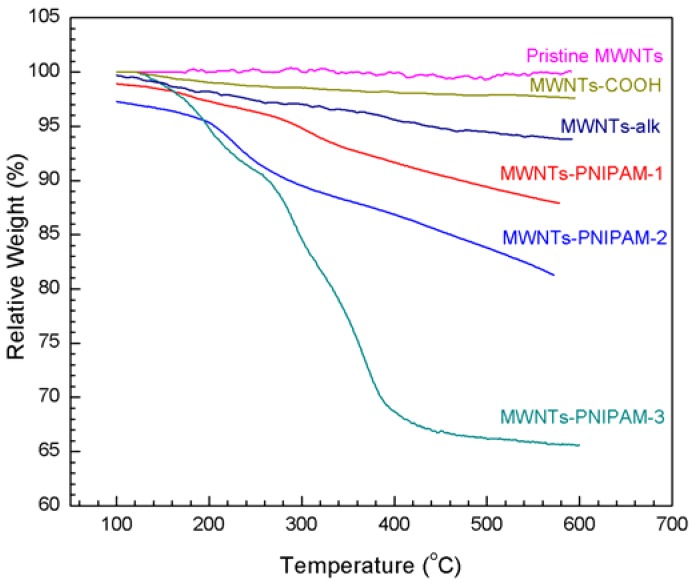
Comparison of the weight losses of pristine MWNTs, MWNTs-COOH, MWNTs-alk, MWNTs-PNIPAM-1, MWNTs-PNIPAM-2 and MWNTs-PNIPAM-3. The data is measured by TGA.

In the present study, the MWNTs were lightly functionalized with nitric acid to afford MWNTs-COOH. MWNTs-COOH were then treated with thionyl chloride and reacted with excess propargyl alcohol to obtain MWNTs-alk where the alkyne groups should be connected on the surface. The MWNTs-alk can be measured via TGA. The TGA results (Figure 1) indicate that the mass losses of MWNTs-COOH and MWNTs-alk were approximately 2.3% and 6.2%, which also shows the alkyne group was successfully introduced. It was found that the amount of alkyne groups introduced into MWNTs is 7.0 × 10^−4^ mol/g, which was calculated by the difference between the mass losses of MWNTs-COOH and MWNTs-alk.

*Reagents and Conditions*: (i) HNO_3_, reflux, 2 h; (ii) SOCl_2_; propargyl alcohol, CHCl_3_, room temperature, 24 h; (iii) NIPAM, AIBN, 1,4-dioxane, 60 °C, 20 h; (iv) CuBr, PMDETA, 70 °C, 12 h.

### 2.2. Synthesis and Characterization of N_3_-PNIPAM

The azide-decorated RAFT agent N_3_-CTA is the most important precursor for obtaining serial N_3_-PNIPAM, and it was obtained by coupling 2-azidoethanol with trithiocarbonate CTA. The ^1^H-NMR spectra of N_3_-CTA showed the following peaks: 0.99 (t, –CH_2_CH_3_, 3H), 1.37–1.47 (m, –CH_2_CH_2_, 20H), 1.75 (s, –C(CH_3_)–CH_3_, 6H), 3.42 (t, –CH_2_S, 2H), 3.5 (t, –CH_2_CH_2_–, 2H), 4.24 (t, –CH_2_CH_2_–, 2H).

After preparation of the azide-capped N_3_-CTA, we can access azide-decorated PNIPAM by RAFT polymerization. It was noticed that increasing the molecular weight of the end-functionalized polymer would inevitably cause a decrease in nanotube grafting efficiency due to the entropy constraints of the polymer and the heterogeneous nature of the grafting reactions [18], but RAFT polymerization is an ideal method to obtain the polymers with controlled degrees of polymerization [16]. Three linear PNIPAM polymers with theoretical M_n_ values of 2.6 × 10^3^, 5.2 × 10^3^ and 11 × 10^3^ g·mol^−1^ and bearing an azide function on one end group were prepared by RAFT polymerization of NIPAM with N_3_-CTA; meanwhile, the reference sample PNIPAM-0 was synthesized by a traditional polymerization method. Their average molecular weights as measured by GPC is reported in Table 1.

**Table 1:** The feed ratio of polymerization and the GPC analysis results of serial N_3_-PNIPAM and PNIPAM-0.

**Entry**	**Sample**	**[M]:[CTA]:[I]**	**M_theo_ (g·mol^−1^)**	**M_n_ (g·mol^−1^)**	**PDI**
1	N_3_-PNIPAM-1	100:5:1	2200	2,180	1.19
2	N_3_-PNIPAM-2	250:5:1	5600	4,790	1.26
3	N_3_-PNIPAM-3	500:5:1	11,000	9,440	1.22
4	PNIPAM-0	200:1 *	/	8,220	2.83

* The PNIPAM-0 as a comparison sample was synthesized by traditional radical polymerization, the reactant didn’t have CTA and 200:1 means the feed ratio of [M]:[I].

**Figure 2:**
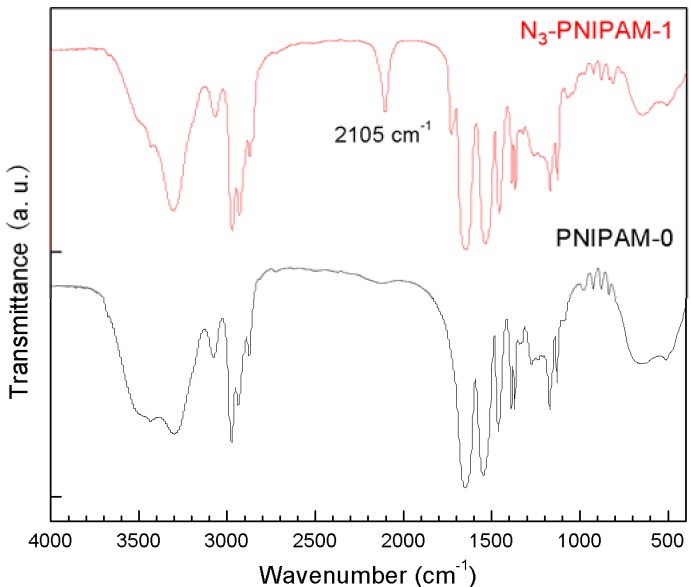
The FT-IR spectra of N_3_-PNIPAM-1and PNIPAM-0.

**Figure 3:**
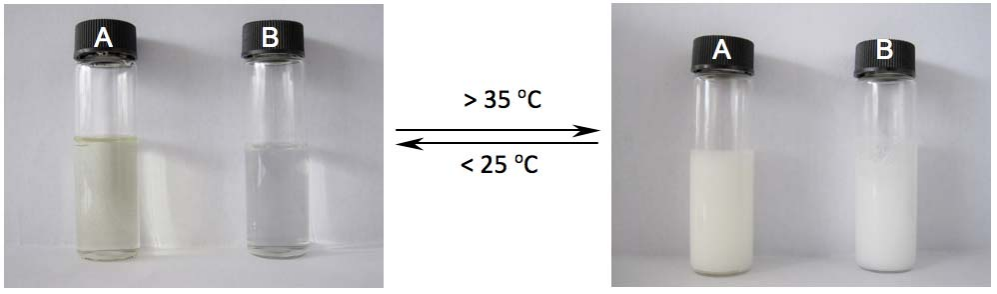
The thermoresponsive performance of N_3_-PNIPAM-1 (**A**) and PNIPAM-0 (**B**) water solution with polymer concentration 2 w.t.%.

The GPC results showed that the molecular weight of the series of N_3_-PNIPAMs obtained by RAFT polymerization was close to the theoretical molecular weight, and the molecular weight distribution was 1.19–1.26, which satisfies the RAFT polymerization criteria, whereas the molecular weight distribution distribution of PNIPAM-0 obtained by traditional free radical polymerization was much wider. Additionally, the N_3_-PNIPAM obtained from RAFT polymerization was a yellow powder, as expected [34,35], whereas the PNIPAM reference sample obtained by traditional polymerization without azide groups was a white powder. Figure 2 compares the FT-IR spectra of N_3_-PNIPAM-1 and PNIPAM-0. It is clear that the N_3_-PNIPAM-1 and the PNIPAM-0 both have characteristic C=O (1,645.7 cm^−1^) and –N(CH_3_)_2_ (1,386 cm^−1^, 1,366 cm^−1^) absorption peaks, but only N_3_-PNIPAM has a peak at 2,105 cm^−1^ which corresponds to the azide absorption, suggesting the N_3_-PNIPAM has the potential ability to connect with MWNTs-alk via azide-alkyne cycloaddition. In order to investigate whether the series of synthesized PNIPAM have temperature-responsive behaviors, the aqueous polymer solutions were first observed visually at different temperatures. The phase transition phenomena of N_3_-PNIPAM-1 and PNIPAM-0 in pure water are compared in Figure 3. At room temperature, the N_3_-PNIPAM solution was light yellow and PNIPAM-0 solution was colorless and transparent. After warming above 35 °C, both solutions immediately became milky white and viscous. 

**Figure 4:**
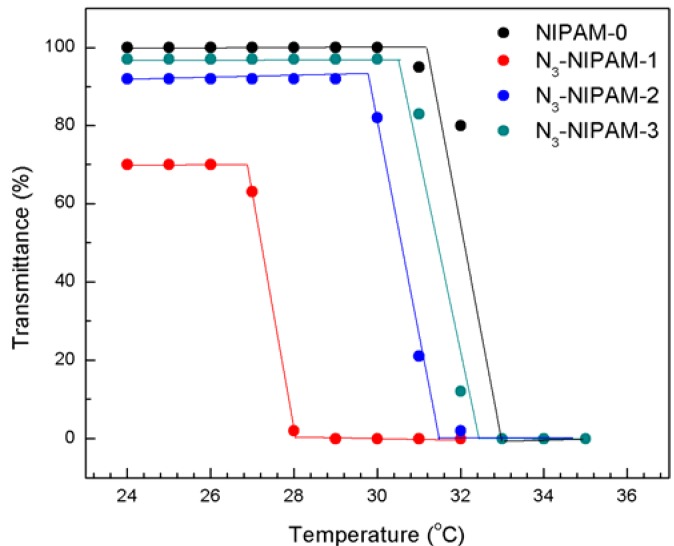
Variation of transmittance with temperature for the series of N_3_-PNIPAM and PNIPAM-0 water solution (2 wt%).

To determine the response temperature accurately, the variation of transmittance with temperature was monitored using an UV-visible spectrophotometer (Figure 4). It was observed that both N_3_-PNIPAM and PNIPAM-0 have thermo-responsive behavior. 

The LCST values of N_3_-PNIPAM-1, N_3_-PNIPAM-2, N_3_-PNIPAM-3 and PNIPAM-0 were 28.5, 30.6, 31.7 and 32 °C, respectively. The N_3_-PNIPAM’s LCST is apparently smaller than the PNIPAM-0, and it can be attributed to the introduction of the hydrophobic chain transfer groups [34]. The difference in LCST for the series of N_3_-PNIPAM polymers was due to the different relative contents of hydrophobic chain transfer agent. When the molecular weight is small, the proportion of CTA hydrophobic head groups was relatively large and the LCST is reduced greatly. In contrast, when the molecular weight was higher, the proportion of the hydrophobic head groups can be ignored, and the LCST was close to pure PNIPAM.

### 2.3. Coupling of Polymers onto MWNTs by Click Reaction

As mentioned previously, it is very quick and simple to connect PNIPAM and MWNTs via click reactions. The cycloadditions between alkyne decorated MWNTs and PNIPAM polymer with azide groups on the outer shell were performed in water in the presence of a Cu(I) catalyst, which was generated *in situ*, resulting in the formation of polymer functionalized MWNTs (Scheme 1). The most attractive features for this coupling are its mild reaction conditions and high reaction efficiency. The reaction was conducted at about 70 °C and the concentration of N_3_-PNIPAM is 3.0 mg·mL^−1^. In this study, the N_3_-PNIPAM is in excess and the remaining polymer can be easily removed after each reaction by ultra-filtration and prolonged washing with water. In addition, trace amounts of copper salts in the products were removed by washing with an aqueous ammonium hydroxide solution and water.

**Figure 5:**
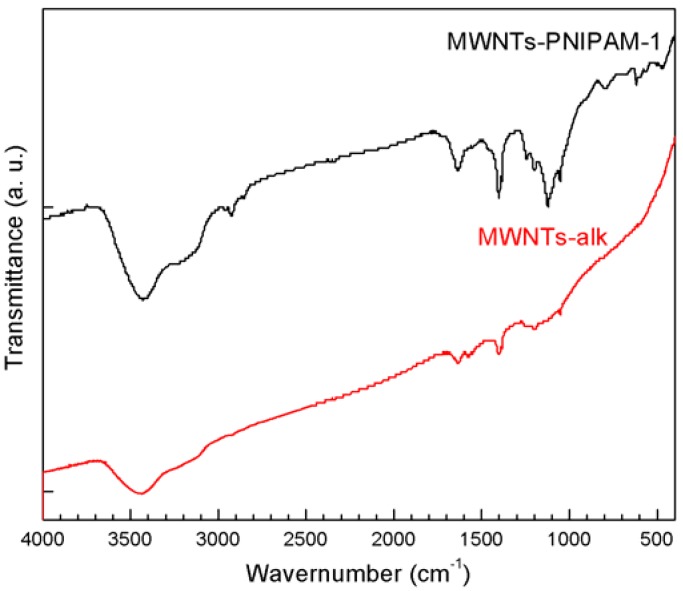
The FT-IR spectra of MWNTs-PNIPAM-1 and MWNTs-alk.

FT-IR spectral comparison between MWNTs-alk and MWNTs-PNIPAM provides information about the structures appended to the surface of MWNTs. The absorbance peaks at 1,620 and 1,743 cm^−1^ can be attributed to C=C, and result from skeletal vibrations of unoxidized graphite domains, C=O in carbonyl moieties, respectively (Figure 5). 

After the coupling reaction, several new peaks appeared in the FT-IR spectrum of MWNTs-PNIPAM, clearly due to the existence of PNIPAM in the products. For example, the characteristic amide group [–C(O)NH–] stretching vibration at 1,555 cm^−1^ indicates the presence of the amide bond originating from PNIPAM. Additionally, there are other bands (1,384.7 cm^−1^ and 1,401 cm^−1^ for –CH(CH_3_)_2_, 1,631 cm^−1^ for C=O, and 3,426 cm^−1^ for –NH), all of which are from the PNIPAM. In short, these results confirmed PNIPAM was successfully grafted onto the MWNTs.

**Figure 6:**
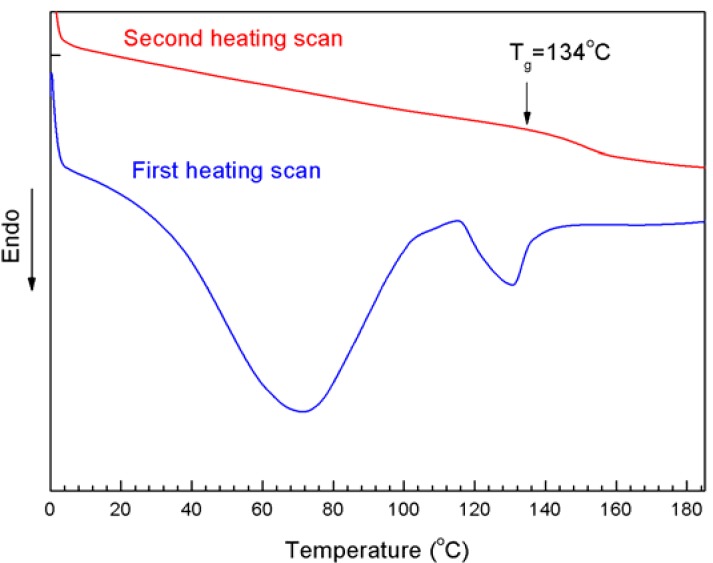
The DSC curves of MWNTs-PNIPAM-1 after twice temperature scan, showing the glass transitions of the complex. Vertical shifts have been used for clarity.

In order to further confirm that the N_3_-PNIPAM was connected onto the MWNTs, a DSC test was performed on the MWNTs-PNIPAM-1 to detect the thermal properties of the surface material (Figure 6). After two temperature scans, it was found its T_g_ was 134 °C, which was also consistent with the T_g_ of PNIPAM from the literature data [35], and the result proved that MWNTs were successfully modified with PNIPAM.

To obtain a quantitative picture of the extent of MWNTs functionalization, thermogravimetric analysis (TGA) was performed on the reaction product (Figure 1). As the samples were washed repeatedly after N_3_-PNIPAM was clicked onto the MWNTs, the physical adsorption and wrapping of polymer chains were eliminated, thus the remaining products were PNIPAM polymer chains grafted on the MWNTs. The molecular weights of the various PNIPAM connected onto MWNTs is 2,180 (N_3_-PNIPAM-1), 4,790 (N_3_-PNIPAM-2) and 9,440 (N_3_-PNIPAM-3) g·mol^−1^, respectively (Table 1). The mass loss of MWNTs-PNIPAM-1, MWNTs-PNIPAM-2 and MWNTs-PNIPAM-3 was approximately 15%, 20% and 34%, due to polymer decomposition, from which we can find around 6.89 × 10^−^^5^ mol of N_3_-PNIPAM-1, 4.17 × 10^−^^5^ mol of N_3_-PNIPAM-1 3.60 × 10^−^^5^ mol of N_3_-PNIPAM-1 were attached onto 1 g of hybrids. The MWNT-PNIPAM-3 showed two major decompositions in the temperature range. One possible reason is that the high molecular weight polymer contains DMF, whose boiling point is 153 °C. The results in Figure 1 show the larger grafted polymer molecular weight indicates the higher rate of weight loss. Nevertheless, the actual measured mass losses of MWNT-PNIPAM are much lower than the theoretical mass losses, which should be 60% (N_3_-PNIPAM-1), 77% (N_3_-PNIPAM-2) and 86% (N_3_-PNIPAM-3). The reason is that only part of the N_3_-PNIPAM was connected on the surface of MWNTs, as some of alkyne groups did not react completely with azide groups. This result suggests that increasing the degree of polymerization has a negative influence on the grafting density for polymers connected on MWNTs, due to steric effects. After modification, there is the intertwining between the molecule of PNIPNAM and MWNTs, and this phenomenon must hinder the connection of azide and alkyne groups. The result indicates that with higher molecular weight PNIPNAM it becomes more difficult to obtain high grafting density.

### 2.4. Dispersion Behavior of MWNTs-PNIPAM in Water

The successful implementation of click reaction on the MWNT surface prompted us to investigate the effect of temperature and block length on the solubility of the resulting MWNTs-polymer conjugates.

**Figure 7:**
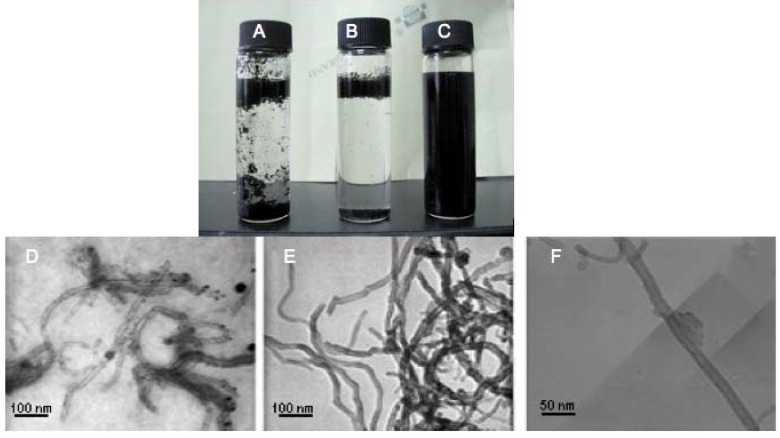
Photographs of (**A**) pristine MWNTs; (**B**) MWNTs-alk and (**C**) MWNTs-PNIPAM-1 in water; (**D**) TEM images of pristine MWNTs; (**E**) MWNTs-alk; (**F**) MWNTs-PNIPAM-1.

The direct qualitative test of MWNTs-PNIPAM is checking the solubility of the modified products in water (Figure 7A–C). It shows the dispersion state of pristine MWNTs, MWNTs-alk and MWNTs-PNIPAM-1 in water at the same concentration (1.0 mg·mL^−1^) after one week at 25 °C, respectively. Clearly, the pristine MWNTs and MWNTs-alk were completely insoluble in water, and the transmittances of supernatant solution measured by UV/Vis spectrophotometer are both higher than 98% at room temperature. Owing to the incorporation of PNIPAM, the PNIPAM-grafted MWNTs can be readily dissolved in pure water by the aid of ultrasonication. The MWNTs-PNIPAM-1 formed a clear, dark-brown solution that exhibits no discernible particulate materials and remained stable for a period of at least four weeks, and the transmittance measured by UV/Vis spectrophotometer is in the range of 3%–5%, even after the suspension had been standing for one month at room temperature. It was found that MWNTs-PNIPAM-1 could be uniformly dispersed in water to afford homogenous solutions that were stable over a long time. The solubility of MWNTs-PNIPAM-1 in pure water was good, due to the benign solubility of PNIPAM, and this also testified to the successful functionalization of MWNTs. The surface of MWNTs turned to hydrophobic from hydrophobic, because of the introduction and modification of the PNIPAM. However, no temperature-response behavior of MWNTs-PNIPAM was observed, perhaps because the graft density is low and there are not enough PNIPAM approaching the surface on MWNTs. The transmission electron microscopy (TEM) images also showed similar results. As shown in Figure 7D, some impurities such as amorphous carbon and metal catalysts were present in the MWNTs. and the MWNTs cannot be dispersed. MWNTs-alk is also intertwined according to the TEM image, but after modification with PNIPAM, the MWNTs can be dispersed individually and completely, indicating that the bundles of original MWNTs were separated into individual tubes by surface modification with polymer chains. Figure 7F shows the highest thickness of the polymer layer on MWNTs is about 20 nm, but only a little amount of PNIPAM is seen on the surface of MWNTs, suggesting a low grafting density. It is known that after hydrophilic functionalization carbon nanotubes are excellent biomedical materials such as a potentially ideal carriers for drug delivery [36]. The click chemistry still supports the possible approach to prepare the hydrophilic functionalized MWNTs. Froms Figure 7D–F, the original diameter of the pristine MWNTs is about 20–30 nm, and there is almost no change in the diameter of MWNTs-alk, but after coupling of PNIPAM and MWNTs, the products resemble partial polymer-encapsulated nanotubes, and the widest part is now more than 50 nm.

## 3. Experimental

### 3.1. Materials

The chain transfer agent S-1-dodecyl-S'-(*α*,*α*'-dimethyl-*α*"-acetic acid)trithiocarbonate (CTA) and 2-azidoethanol were synthesized in our lab according to a previously reported procedure [37,38]. The pristine MWNTs (purity 90%, 20–30 nm in diameter and several micrometers in length (Timesnano Inc., Chengdu, China) were used without further purification. *N*-isopropylacrylamide (NIPAM, 97%, Tokyo Kasei Kagyo, Japan) was recrystallized with toluene/hexane (1/6, v/v) prior to use. *N*,*N*,*N*',*N*",*N*"-pentmethyldiethylenetriamine (PMDETA, 98%), CuBr (99.999%), 1,3-dicyclohexyl-carbodiimide (DCC) and 4-(dimethylamino)pyridine (DMAP) were obtained from Sigma Aldrich (Milwaukee, WI, USA). Other reagents or analytical grade solvents were from commercial resources.

### 3.2. Synthesis of Alkyne-Modified Multi-Walled Carbon Nanotubes (MWNTs-alk)

Typically, pristine MWNTs (1.0 g) were added to an aqueous HNO_3_ solution (60 wt%, 20.0 mL). The mixture was treated in a sonication bath (40 kHz) for 10 min and stirred for 2 h at reflux, then filtered through a poly(tetrafluoroethylene) (PTFE) membrane and washed with distilled water until the pH of the filtrate was approximately 7.0. The treated MWNTs were dried under vacuum for 28 h at 40 °C, affording 0.95 g of carboxyl-functionalized MWNTs (MWNTs-COOH).

The obtained MWNTs-COOH (0.10 g) was suspended in SOCl_2_ (2 mL) in a 10 mL round-bottomed flask and this suspension was stirred at 65 °C for 24 h. After the excess SOCl_2_ was removed under reduced pressure, the flask was cooled in an ice bath. A mixed solution of propargyl alcohol (1 mL, 16.9 mmol), CHCl_3_ (2 mL), and anhydrous triethylamine (1 mL, 7.17 mmol) was added dropwise into the flask over a period of 0.5 h under magnetic stirring; the mixture was stirred at 0 °C for 1 h and then at room temperature for 24 h. The product was filtered through a PTFE membrane under vacuum, and the filter cake was washed with THF and distilled water for several times. The obtained MWNTs-alk was dried under vacuum at 25 °C for 48 h.

### 3.3. Synthesis of N_3_-PNIPAM

Firstly, trithiocarbonate CTA was synthesized following the previously reported procedure [37]. The azide decorated RAFT agent, N_3_-CTA, was prepared by coupling 2-azidoethanol with the trithiocarbonate CTA in the presence of DCC and DMAP in dry CH_2_Cl_2_. A typical procedure was as follows: trithiocarbonate CTA (2.00 g, 5.46 mmol), DDC (1.69 g, 8.19 mmol), DMAP (1.00 g, 5.46 mmol), and dichloromethane (10 mL) were placedd in a round-bottomed flask and stirred for 10 min under an inert atmosphere. 2-Azidoethanol (1 mL, 17.8 mmol) was added and the mixture was stirred overnight at room temperature. The N_3_-CTA product was precipitated in diethyl ether, filtered and dried at room temperature. Next, the N_3_-CTA was washed with acidic water, brine and water successively and finally dried under reduced pressure. The yield was 50.5%. After the N_3_-CTA was obtained, PNIPAM was synthesized by RAFT polymerization of the corresponding monomer with N_3_-CTA as the initiator. NIPAM (1.0 g, 8 mmol), AIBN (0.013 g, 0.08 mmol), N_3_-CTA (0.173 g, 0.4 mmol) and 1,4-dioxane (10 mL) were added to a round-bottomed flask; the mixture in the flask was deoxygenated by a freeze-pump-thaw process, and the reaction was then maintained for 20 h at 60 °C in an oil bath.

### 3.4. Coupling of N_3_-PNIPAM and MWNTs-alk via Click Reaction

In a typical experiment, MWNTs-alk (12.0 mg) were dispersed in DMF (15 mL) by sonication for 5 min and bubbling with nitrogen for 10 min, then N_3_-PNIPAM (1.00 g), CuBr (29 mg, 0.2 mmol) and PMDETA (4 μL, 0.2 mmol) were added. The reaction vessel was evacuated and refilled with nitrogen three times, followed by stirring under nitrogen at 70 °C for 12 h. The reaction was terminated by cooling the reaction flask in an ice bath followed by exposure to air. The mixture was diluted with DMF (10 mL), sonicated for 5 min, and filtered through a PTFE membrane, then washed with THF, aqueous ammonium hydroxide solution and pure water twice, respectively.

### 3.5. Characterizations

The structure of N_3_-CTA was characterized by FT-IR (KBr, Nicolet 6700, Thermo Fisher Scientific, Waltham, MA, USA). The molecular weight and PDI of the polymers prepared were determined by gel permeation chromatography (GPC) using a Waters 515 pump (Waters, Milford, MA, USA), Ohpak KB-803 column (Showa Denko America, Inc., New York, NY, USA) and Waters 2410 refractive-index (RI) detector. The equipment was calibrated with poly(ethylene oxide) (PEO) standards, and phosphate buffer (pH = 6.5) at 1.0 mL·min^−1^ was used as the eluent at a flow rate of 0.80 mL·min^−1^. The phase-transition temperature of N_3_-PNIPAM was determined on a U-2010 UV-visible spectrophotometer (Hitachi, Tokyo, Japan) with controlled temperature. Thermal decomposition of MWNTs-PNIPAM was studied by TGA with a TG Q500 thermal analysis system (TA Instruments Inc., New Castle, DE, USA), whereby TGA scans were recorded at 10 °C·min^−1^ under a N_2_ atmosphere from 100 °C to 600 °C. The thermal characterization studies were conducted by DSC with an EXSTAR 6000 (SII NanoTechnology Inc., Tokyo, Japan), in which the heating rate was 10 °C·min^−1^. The structural morphology of the MWNTs-PNIPAM was observed using an H-6100IV (Hitachi) transmission electron microscopy (TEM) at an acceleration voltage of 120 Kv.

The solubility of the series of PNIPAMs in water was measured by centrifugation and the UV/Vis spectrophotometer. A vial charged with MWNTs-PNIPAM (10 mg) and water (10 mL) was sonicated for 10 min. Then the vial was centrifuged at 4,000 rpm for 10 min and subsequently allowed to stand undisturbed overnight. The supernatant was carefully separated and diluted with water to appropriate concentrations for UV/Vis absorption measurement at certain temperatures.

## 4. Conclusions

We have demonstrated an efficient approach to functionalizing MWNTs with a well-defined PNIPAM via click chemistry. TGA, FI-IR and DSC results proved the successful coupling between N_3_-PNIPAM and MWNTs-alk. MWNTs-PNIPAM has good solubility in pure water and no temperature-response behavior was observed, presumably because of low graft density. It is believed that, with their good solubility and stability in water, the MWNTs-PNIPAM represent a potential nano-material for the preparation of novel composite materials, and click chemistry is one of possible and promising functionalization methods for MWNTs.

## Selected Abbreviations and Acronyms

CNTs: Carbon nanotubes
MWNTs: Multi-walled carbon nanotubes
SWNTs: Single-walled carbon nanotubes
PNIPAM: poly(*N*-isopropylacrylamide)
N_3_-CTA: Azide-capped chain transfer agent
N_3_-PNIPAM: Azide-terminated poly(*N*-isopropylacrylamide)
MWNTs-alk: MWNTs with alkyne groups
MWNTs-PNIPAM: MWNTs functionalized by N3-PNIPAM
